# TSLP and TSLP receptors variants are associated with smoking

**DOI:** 10.1002/mgg3.842

**Published:** 2019-07-09

**Authors:** Abdelhabib Semlali, Mikhlid Almutairi, Arezki Azzi, Narasimha Reddy Parine, Abdullah AlAmri, Saleh Alsulami, Talal Meshal Alumri, Mohammad Saud Alanazi, Mahmoud Rouabhia

**Affiliations:** ^1^ Groupe de Recherche en Écologie Buccale, Département de stomatologie, Faculté de Médecine Dentaire Université Laval Québec Québec Canada; ^2^ Department of Biochemistry College of Science King Saud University Riyadh Kingdom of Saudi Arabia; ^3^ Zoology Department College of Science King Saud University Riyadh Kingdom of Saudi Arabia; ^4^ Department of Biochemistry, College of Medicine Imam Mohammad Ibn Saud Islamic University (IMSIU) Riyadh Kingdom of Saudi Arabia

**Keywords:** genotyping, innate immunity and inflammation, polymorphism, smoking, TSLP pathway

## Abstract

**Background:**

To search for new prevention markers for early detection of the diseases caused by tobacco, we aimed to investigate the polymorphisms in TSLP and TSLPRs associated with cigarette smoking in the Saudi population.

**Materials and methods:**

Samples were collected from 177 smokers and 126 healthy controls. Three TSLP SNPs [rs3806933, rs2289276, and rs10043985], three TSLPR SNPs [rs36133495, rs36177645, and rs36139698], and two IL7R SNPs rs1053496 and rs12516866 were analyzed by genotyping.

**Results:**

Two TSLP SNPs (rs10043985 and rs3806933) and one TSLPR SNP (rs36139698) showed significant correlations with smoking behavior, but not IL7R rs12516866 and rs1053496. rs10043985 showed a clear association with long‐term smoking regardless of daily cigarette consumption. rs2289276 was associated with short‐term smoking but not with daily cigarette consumption. rs3806933 was highly associated with different smoker subgroups. Rs36139698 was highly associated with long‐term smokers who consumed ≥20 cigarettes/day, and the “T” allele was associated only with individuals who smoked ≤20 cigarettes/day. Rs36139698 corresponds to a P195L substitution and produces a TSLPR mutant with a predicted ΔΔG increase of 2.15 kcal/mol and has a more stable structure than the wild‐type variant.

**Conclusions:**

Investigating TSLP and TSLPR polymorphisms is crucial for elucidating the mechanisms underlying tobacco‐induced diseases.

## 1 INTRODUCTION

Environmental pollutants, such as tobacco smoke, support an immune milieu that promotes allergic asthma (Spann, Snape, Baturcam, & Fantino, 2016). Individuals with long‐term cigarette consumption have substantially increased risk of developing asthma, chronic obstructive pulmonary disease (COPD), and oral and lung cancers (Centers for Disease Control & Prevention, 2010). Cigarette smoking (CS) is a major public health concern that causes a global increase in mortality rates and vulnerability to certain diseases (Baig et al., 2016). It was estimated that globally, there are currently 1.2 billion smokers over the age of 15 years WHO (2018). According to the WHO report in 2016, smoking is associated with around 6 million deaths per year worldwide. More than 5 million of these are caused by direct tobacco use, and over 600,000 are due to exposure to secondhand smoke. In the Kingdom of Saudi Arabia (KSA), the incidence of CS in certain regions is greater than 50% (Bassiony, 2009). Based on the WHO analyses, 3 million KSA residents were smokers in 2010; however, this number is predicted to increase to around 6 million by 2025. The above findings have prompted government agencies to increase public awareness on the health risks of tobacco use. A wide variety of diseases are attributed to smoking (Qiu et al., (2017)). In developed countries, CS is responsible for ~30% of all cancer mortalities and morbidities, most of which are attributed to lung cancer (ACS Inc, 2014; Gutierrez, Suh, Abtin, Genshaft, & Brown, 2013) and diseases affecting the cardiovascular system (Menotti, Puddu, Maiani, & Catasta, 2015). Previous reports by Alamri et al. (2015). have also emphasized the role of tobacco in causing damage to gingival cells. In particular, CS deregulates multiple cell functions, including growth (Alamri et al., 2015), adhesion, and migration (Semlali, Chakir, Goulet, Chmielewski, & Rouabhia, 2011), which have been observed in fibroblasts and human gingival epithelial cells (Semlali, Chakir, Goulet, et al., 2011; Semlali, Chakir, & Rouabhia, 2011). In addition, CS has been reported to promote apoptosis in epithelial cells and impair the cell repair process (Semlali, Chakir, & Rouabhia, 2011). Multiple chemical and biological studies have also revealed the harmful effects of many tobacco components, which have been particularly demonstrated to influence mutagenesis and DNA methylation (Steenaard et al., 2015) and induce genetic alterations in pro‐oncogenes and tumor suppressor genes, as well as p53 (HusgafvelPursiainen & Kannio, 1996; Pfeifer et al., 2002; Taghavi et al., 2010) and innate immunity genes (Kohailan et al., 2017, 2016).

Previous studies have clearly demonstrated that CS induces chronic inflammation in the conducting airways through multiple mechanisms. Direct activation of immune cells induces the secretion of proinflammatory factors, as well as IL‐6, TNF‐α, and TSLP (thymic stromal lymphopoietin).

TSLP is an interleukin 7 (IL‐7)‐like cytokine secreted primarily by human bronchial epithelial cells (Liu et al., 2011). TSLP has been recognized as a primary instigator of allergic inflammation at the dendritic and epithelial cell interface (Liu et al., 2007) and has been shown to play an important role in innate immune response by inducing the differentiation of T‐helper type 2 (Th2) effector cells in asthma patients. Various protease allergens, respiratory viruses, and inflammatory cytokines are known to induce TLSP upregulation in airway epithelial cells (Tsilingiri, Fornasa, & Rescigno, 2017; Ziegler & Artis, 2010). The human *TSLP* is located on chromosome 5q22.1 and is adjacent to the gene cluster that encodes Th2 cytokines (Quentmeier et al., 2001). TSLP comprises the TSLP receptor (TSLPR) and interleukin 7 receptor (IL7R) alpha chain (Pandey et al., 2000; Park et al., 2000). TSLPR is a novel receptor subunit that forms the receptor for TSLP in conjunction as a heterodimeric complex with the IL7R alpha chain (Pandey et al., 2000). Like all cytokine receptors, the TSLPR subunit has a conserved WSXWS (Trp‐Ser‐X‐Trp‐Ser) motif in the extracellular domain; however, its role is not precisely understood (Hilton, Watowich, Katz, & Lodish, 1996; Tonozuka et al., 2001; Zhang et al., 2001). Knockout experiments in mice have demonstrated that TSLPR plays a crucial role in the lung inflammatory response and/or allergic responses (Al‐Shami, Spolski, Kelly, Keane‐Myers, & Leonard, 2005). Recently, Shi et al. suggested that local inhibition of TSLPR alleviated allergic responses by regulating the function of dendritic cells (DCs) (Shi et al., 2008). Furthermore, a recent study indicated *TSLP* as a strong susceptibility gene for asthma among adult Japanese populations (Harada et al., 2011). TLSP is strongly expressed in the submucosa and bronchial epithelia of clinically stable asthmatic patients and is also correlated with airway obstruction (Ying et al., 2005). Recently, it was proved that cigarette smoke induces TSLP expression, leading to T(H)2‐type immune responses and airway inflammation.

Recent studies provided evidence that CS induces further genetic alterations, such as single nucleotide polymorphisms (SNPs), in innate immunity genes (Kohailan et al., 2016) that can in turn lead to a range of diseases (Steenaard et al., 2015) or induce transitions or transversions (Acevedo, Brodsky, & Andino, 2014; Farrell et al., 2014). One study found significant correlations between genetic variants of *TSLP* and asthma (Liu et al., 2011). Another study showed that the rs1837253 SNP, which is located 5.7 kb upstream of the *TSLP* transcription start site, was linked to asthma in a Canadian population (He et al., 2009). Furthermore, significant differences in the genotypes and allele frequencies of *TSLPR* were found between asthmatic patients and healthy controls in a Korean population (Semlali, Parine, et al., 2017). We hypothesized that the development of smoking‐induced respiratory and cancer diseases is mediated by genetic changes in the genes encoding TSLP and TSLP receptors (TSLPR and IL7R). Interestingly, no previous studies have investigated the relationship between smoking and the SNPs in these three genes. Thus, the present study aimed to determine whether genetic variants in *TSLP* (rs3806933, rs2289276, and rs10043985), *TSLPR* (rs36133495, rs36177645, and rs36139698), and *IL7R* (rs1053496 and rs12516866) are associated with cigarette smoking in Saudi Arabians. The SNPs studied were selected based on their known involvement in various diseases, which could be explained by their ability to alter gene function and to ultimately influence the pathogenesis of other unstudied diseases.

## 2 MATERIALS AND METHODS

### 2.1 Ethics statement and sample collection

All methods were carried out in accordance with relevant guidelines and regulations and all experimental protocols were approved by a Research Ethics Committee of the College of Applied Medical Sciences at King Saud University (KSU) in Riyadh, Saudi Arabia (Approval Number: CAMS 13/3536). In this sense, written ethical consent for this study was reviewed by and obtained from this Research Ethics Committee of the College of Applied Medical Sciences at King Saud University (KSU). Participants who smoked cigarettes were termed smokers, whereas individuals who did not consume any kind of tobacco product were referred to as nonsmokers. Smokers were divided into two groups based on cigarette consumption, namely, those who smoked ≥20 cigarettes/day and those who smoked <20 cigarettes/day. All volunteer smokers and nonsmokers signed a written informed consent. Clinical data on smoking history, allergic symptoms and diseases, number of cigarettes smoked daily, and body mass index (BMI) were obtained through a self‐completed questionnaire.

Saliva samples were collected from a group of 177 cigarette smokers (smokers) and a group of 126 healthy controls (nonsmokers) recruited from academic staff and only male students at KSU between January 2015 and April 2015. Participating volunteers were not suffering from any diseases or disorders. Detailed clinical characteristics of the participants are summarized in Table 1.

**Table 1:** Clinical and demographic data of the Saudi population included in the study

Variable	Smokers	Nonsmokers
Number	177	126
Age (years), median ± average	24 ± 27	20 ± 21
BMI		
Obese (≥30 kg/m^2^)	27/163 (17%)	20/100 (20%)
Nonobese (˂30 kg/m^2^)	136/163 (83%)	80/100 (80%)
Years of smoking		
>5	104/165 (63%)	—
≤5	61/165 (37%)	—
Daily cigarette		
≥20	99/159 (62.3%)	—
˂20	60/159 (37.7%)	—

Abbreviation: BMI, body mass index.

### 2.2 DNA extraction

DNA extraction was performed as previously described (Kohailan et al., 2017, 2016; Semlali, Jalouli, et al., 2017; Semlali, Parine, et al., 2017; Semlali et al., 2016). Briefly, saliva samples were diluted twice in phosphate‐buffered saline, and DNA was isolated using the PureLink^®^ Genomic DNA Mini Kit (Catalog No K1820‐01; Invitrogen™, Carlsbad, CA) according to the manufacturer's instructions. DNA concentration was quantitated using a NanoDrop 8000 (Thermo Fisher Scientific, Waltham, MA) instrument, and DNA purity was determined by calculating the A_260 nm_/A_280 nm_ and A_260 nm_/A_230 nm_ ratios.

### 2.3 Candidate SNP selection and TaqMan genotyping assay

10 ng/ul of each genomic DNA collected from saliva was used for genotyping. Eight tagged SNPs in *TSLP* and *TSLPR* were used in this study. Three SNPs in *TSLP* (rs3806933 [1350T/C, Ser450Ser], rs2289276 [1350T/C, Ser450Ser], and rs10043985 [597T/C, Asn199Asn]), three SNPs in *TSLPR* (rs36133495 [1350T/C, Ser450Ser], rs36177645 [1350T/C, Ser450Ser], and rs36139698 [597T/C, Asn199Asn]), and two SNPs in *IL7R* (rs1053496 [979 G/A, Val327Met] and rs12516866 [745T/C, Ser249Pro]) were selected based on their locations in the gene regulatory regions. All SNPs were located either in the promoter regions, 5'‐untranslated regions (5'‐UTR), or exons (Table 2). These SNPs were also selected based on literature reviews of SNP associations with various diseases in diverse ethnic groups. Each genotyping reaction contained 0.2 µl of 40× TaqMan® Genotyping SNP Assay (Applied Biosystems), 5.6 µl of TaqMan^®^ Genotyping Master Mix (Applied Biosystems, Foster City, CA), and 20 ng of DNA. Reactions were run on a QuantStudio™ 7 Flex Real‐Time PCR System (Applied Biosystems) with an end point reading of the genotypes (Semlali, Jalouli, et al., 2017; Semlali, Parine, et al., 2017).

**Table 2:** Description of the selected SNPs

Gene	SNP ID	SNP location	Variation type	Amino acid/nucleotide change	Alleles change
*TSLP*	rs3806933	NC_000005.10:g.111071044	Promoter		C/T
	rs2289276	NC_000005.10:g.111071809	5'‐UTR		C/T
	rs10043985	NC_000005.10:g.111065770	Promoter		A/C
*TSLPR*	rs36133495		Exon	C/T(A238V)	C/T
	rs36177645		Exon	A/G(X210W)	A/G
	rs36139698		Exon	C/T(P196L)	C/T
*IL‐7R*	rs1053496	NC_000005.10:g.35879327	3'‐UTR		C/T
	rs12516866	NC_000005.10:g.35851159	Promoter		G/T

### 2.4 Data analysis

As described in our previous work (Semlali, Jalouli, et al., 2017; Semlali, Parine, et al., 2017), the calculated genotypic and allelic frequencies of each SNP were checked for the Hardy‐Weinberg equilibrium deviation. Genetic comparisons were performed using the χ^2^ test and calculation of allelic odds ratios (ORs). In addition, 95% confidence intervals (CIs) were determined using Fisher's exact test (two‐tailed). All statistical analyses were performed using Statistical Package for the Social Sciences (SPSS) version 16.0 statistical software (SPSS, Chicago, USA). *p* < 0.05 was considered statistically significant.

Homology modeling of the 3D structure of the human TSLPR was performed on the SWISS‐MODEL server using the X‐ray structure of the mouse TSLPR included in the TSLPN123Q–TSLPRN53Q‐IL7Rα complex (Protein Data Bank entry 4NN7) (Verstraete et al., 2014), with which it shares 35% sequence identity, as a model.

The resulting homology model of the human TLSPR was used to estimate the impact of the selected mutations on protein structure. Changes in thermal protein stability for the rs36139698 mutant was predicted using the CUPSAT stability prediction server (Parthiban, Gromiha, & Schomburg, 2006), which evaluates the changes in free energy during the protein folding‐unfolding process (the ΔΔ*G*) as a result of the mutation. A positive or negative ΔΔ*G* value indicates that the mutation is thermodynamically stabilizing or destabilizing, respectively, while the magnitude of ΔΔ*G* is a measure of the extent of the alteration.

## 3 RESULTS

### 3.1 General clinical patient characteristics

A total of 177 smoker patients and 126 nonsmoker controls from the Saudi Arabian population were included in the present study. The clinical and the demographic characteristics of the study population are described in Table 1. Our analysis revealed no significant differences in BMI and age between smoking and nonsmoking individuals (Table 1). The average ages for both groups were 20 ± 21 years for nonsmokers and 24 ± 27 years for smokers; 17% of nonsmokers and 20% of smokers were suffering from obesity. The smoker group was divided into two subgroups based on duration of smoking, namely, individuals who smoked for >5 years, which comprised 63% of all smokers, and individuals who had smoked for ≤5 years, which comprised 37% of all smokers. The smoker subgroups were further classified into two categories according to the number of cigarettes smoked daily, namely, smokers who consumed ≥20 cigarettes (one pack of cigarettes) daily and those who consumed <20 cigarettes daily (Table 1).

### 3.2 Genotypic patterns of *TSLP*, *TSLPR*, and *IL7R* SNPs among smokers and nonsmokers

In this study, we collected a total of 177 samples from smokers and 126 samples from nonsmokers and studied the association of genetic variants in *TSLP*, *TSLPR*, and *IL7R* with smoking behavior. A general comparison between the genotype distribution and allele frequencies between smokers and controls for the eight tested SNPs are described in Table 3. Only rs10043985 and rs3806933 showed statistically significant correlations with smoking behavior. For rs10043985, the genotypic distribution was 84% AA, 7% AC, and 9% CC in nonsmokers and 79% AA, 21% AC, and 0.65% CC in smokers (*p* < 0.05). In particular, “AC” heterozygous allele showed around one third higher correlation with smoking than the homozygous “AA” allele (OR = 3.44; CI = 1.278–9.248; *p* = 0.0103). The homozygous “CC” allele was found to be significantly correlated with smoking (OR = 0.08; CI = 0.009–0.637; *p* < 0.005), and the allele distribution was similar between smokers and controls (*p* = 0.679). For rs3806933, smoker groups and control groups showed significant differences in genotype frequencies of “CT”, “TT,” and “CT + TT” (*p* < 0.005) when compared to the wild‐type “CC” genotype. In addition, the “T” allele showed a significant phenotypic correlation with smoking individuals when compared to the “C” reference allele. The phenotypic distribution was 33% C and 67% T in normal controls and 45% C and 55% T in smokers (*p* = 0.0075). By contrast, rs2289276 showed similar genotype and allele frequencies between smokers and controls (Table 3).

**Table 3:** Genotypic allocations of TSLP, TSLPR, and IL‐7R gene polymorphisms among smokers and controls

Gene	SNP	Alleles	Controls			Smokers		OR	95% CI	χ^2^	*p* value
			*N*		Percent	*N*	Percent				
TSLP	rs10043985	total	77		100	154	100				
		AA	65		84	121	79	Ref			
		AC	5		7	32	21	3.44	1.2781–9.2484	6.5824	0.0103*
		CC	7		9	1	0	0.08	0.0092–0.6374	9.0765	<0.005*
		AC+CC	12		16	33	21	1.48	0.7146–3.0539	1.1177	0.2904
		A	135		88	274	89	Ref			
		C	19		12	34	11	0.88	0.4848–1.6034	0.1705	0.6797
	rs2289276	total	113		100	167	100				
		CC	45		40	62	37	Ref			
		CT	56		49	82	49	1.06	0.6365–1.7745	0.0542	0.8159
		TT	12		11	23	14	1.39	0.6272–3.0853	0.6627	0.4156
		CT+TT	68		60	105	63	1.12	0.6864–1.8300	0.2077	0.6486
		C	146		65	206	62	Ref			
		T	80		35	128	38	1.13	0.7985–1.6103	0.4940	0.4821
	rs3806933	total	98		100	124	100				
		CC	6		6	48	39	Ref			
		CT	52		53	16	13	0.04	0.0139–0.1063	51.5551	<0.005*
		TT	40		41	60	48	0.19	0.0734–0.4792	13.9699	<0.005*
		CT+TT	92		94	76	61	0.10	0.0419–0.2543	31.5786	<0.005*
		C	64		33	112	45	Ref			
		T	132		67	136	56	0.59	0.3988–0.8691	7.1587	0.0075*
TSLPR	rs36139698	total	55		100	131	100				
		CC	6		11	8	6	Ref			
		CT	11		20	65	50	4.43	1.2871–15.2604	6.2165	0.0127*
		TT	38		69	58	44	1.14	0.3680–3.5608	0.0546	0.8153
		CT+TT	49		89	123	94	1.88	0.6210–5.7075	1.2834	0.2573
		C	23		21	81	31	Ref			
		T	87		79	181	69	0.59	0.3481–1.0026	3.8519	0.0497*
	rs36177645	total	119		100	93	100				
		AA	13		11	10	11	Ref			
		AG	39		33	39	42	1.30	0.5097–3.3157	0.3025	0.5823
		GG	67		56	44	47	0.85	0.3444–2.1165	0.1167	0.7327
		AG+GG	106		89	83	89	1.02	0.4252–2.4371	0.0016	0.9682
		A	65		27	59	32	Ref			
		G	173		73	127	68	0.81	0.5312–1.2313	0.9811	0.3219
	rs36133495	total	119		100	157	100				
		CC	24		20	32	20	Ref			
		CT	61		51	79	50	0.97	0.5194–1.8162	0.0083	0.9274
		TT	34		29	46	30	1.01	0.5088–2.0238	0.0017	0.9669
		CT+TT	95		80	125	80	0.99	0.5456–1.7851	0.0019	0.9651
		C	109		46	143	46	Ref			
		T	129		54	171	54	1.01	0.7205–1.4170	0.0036	0.9521
IL‐7R	rs12516866	total	123	100		64	100				
		GG	49	40		31	48	Ref			
		GT	61	50		28	43	0.73	0.3847–1.3683	0.9851	0.3209
		TT	13	10		5	9	0.61	0.1973–1.8730	0.7612	0.3830
		GT+TT	74	60		33	52	0.70	0.3835–1.2957	1.2719	0.2594
		G	159	65		90	70	Ref			
		T	87	35		38	30	0.77	0.4869–1.2230	1.2200	0.2694
	rs1053496	total	89	100		56	100				
		CC	11	12		7	12	Ref			
		CT	19	22		15	27	1.24	0.3871–3.9757	0.1318	0.7165
		TT	59	66		34	61	0.91	0.3209–2.5553	0.0351	0.8513
		CT+TT	78	88		49	88	0.99	0.3586–2.7179	0.0006	0.9801
		C	41	23		29	26	Ref			
		T	137	77		83	74	0.86	0.4951–1.4819	0.3069	0.5796

* *p* < 0.05, Ref = Reference allele.

The observed genotype frequency distribution for *TSLPR* revealed that out of the three SNPs tested, only rs36139698 exhibited significant differences between smoker and nonsmokers, with OR = 4.43 and *p* = 0.04. However, the genotype distributions were 11% CC, 20% CT, and 69% TT in nonsmokers and 6% CC, 50% CT, and 44% TT in smokers. The “CT” heterozygous allele showed around 25% higher correlation with smoking than the “CC” homozygous allele (OR = 4.43; CI = 1.287–15.260; *p* = 0.0127). Notably, an association was found between the “T” allele in rs36139698 and smoking when compared to the “C” allele (OR = 0.59; CI = 0.348–1.003; *p* = 0.0497) (Table 3). In addition, the genotype and allele frequencies for rs36177645 and rs36133495 in *TSLPR* did not appear to be influenced by cigarette smoking (Table 3).

Finally, our results showed no statistically significant correlations between smoking and the IL7R SNPs rs12516866 and rs1053496. For *IL7R,* the genotype frequencies for rs12516866 were 40% GG, 50% GT, and 10% TT in nonsmokers and 48% GG, 44% GT, and 8% TT in smokers. On the other hand, the genotypes frequencies in the IL7R rs1053496 SNP were 12% CC, 22% CT, and 66% TT in nonsmokers and 12% CC, 27% CT, and 61% TT in smokers (Table 3).

### 3.3 Association of gene polymorphisms of *TSLP*, *TSLPR*, and *IL7R* with duration of smoking

As mentioned earlier, patients in the present study were classified into the following two categories based on smoking duration: long‐term smokers, which included individuals who had been smoking for >5 years, and short‐term smokers, which included individuals who had smoked for ≤5 years. Table 4 shows the statistical analyses and genotype distributions for the *TSLP*, *TSLPR*, and *IL7R* variants for each subgroup when compared with the nonsmoking individuals. Analysis of the genotype distributions and allele frequencies for *TSLP* showed that rs10043985 results in a fourfold higher risk for developing cigarette‐associated diseases in long‐term smokers but not in short‐term smokers when compared to nonsmokers. In addition, the genotype frequency of “AC” was 7% in controls and 24% in long‐term smokers (*p* < 0.005). However, “AC” genotype frequencies were not statistically significant between nonsmokers and short‐term smokers (Table 4). Conversely, the *TSLP* rs2289176 variant was clearly more highly associated with short‐term smokers compared to control subjects by approximately 3.75 times but was not associated with long‐term smokers. The genotype and allele frequencies for rs2289176 were 22%, 9%, and 11% for short‐term smokers, long‐term smokers, and nonsmoker subjects, respectively, for the homozygote genotype TT” and 50%, 32%, and 35% for the “T” allele (Table 4). For *TSLP* rs3806933, the “TT” genotype displayed a significant association with smoking in the two smoker subgroups relative to nonsmoker patients. In addition, “CT,” “TT,” and combined “CT+TT” genotypes appeared to exhibit significant associations relative to the “CC” homozygous reference allele in both long‐term (OR = 0.04, CI = 0.012–0.124, *p* = 0.005; OR = 0.22, CI = 0.081–0.592, *p* = 0.005; and OR = 0.12, CI = 0.045–0.306, *p* = 0.005, respectively) and short‐term smokers (OR = 0.04, CI = 0.012–0.143, *p* = 0.005; OR = 0.17, CI = 0.057–0.493, *p* = 0.005; and OR = 0.10, CI = 0.034–0.269, *p* = 0.005, respectively). However, the “T” allele showed a significant association with smoking only in short‐term smokers (*p* = 0.0172) but not in long‐term smokers relative to the “C” allele for both the short‐term (OR = 0.53; CI = 0.316–0.899; *p* = 0.0175) and long‐term smokers (OR = 0.68; CI = 0.4333–1.0534; *p* = 0.0830) (Table 4).

**Table 4:** Comparison of genotypic distributions of TSLP, TSLPR, and IL‐7R gene SNPs in smokers with entire controls based on duration of smoking

Gene	SNP	Allele	Controls		>5 years		OR	95% CI	χ^2^	*p* value
			*N*	Percent	*N*	Percent				
Patients smoking for >5 years										
TSLP	rs10043985	total	77	100	88	100				
		AA	65	84	67	76	Ref			
		AC	5	7	21	24	4.07	1.4499–11.4509	7.9287	<0.005*
		CC	7	9	0	0	—	—	6.8593	0.0088*
		AC+CC	12	16	21	24	1.70	0.7728–3.7300	1.7593	0.1847
		A	135	88	155	88	Ref			
		C	19	12	21	12	0.96	0.4965–1.8664	0.0127	0.9103
	rs2289276	total	113	100	97	100				
		CC	45	40	43	44	Ref			
		CT	56	49	45	47	0.84	0.4740–1.4919	0.3510	0.5536
		TT	12	11	9	9	0.78	0.3005–2.0500	0.2452	0.6205
		CT+TT	68	60	54	56	0.83	0.4795–1.4402	0.4355	0.5093
		C	146	65	131	68	Ref			
		T	80	35	63	32	0.88	0.5849–1.3169	0.3975	0.5284
	rs3806933	total	98	100	73	100				
		CC	6	6	26	36	Ref			
		CT	52	53	9	12	0.04	0.0128–0.1243	39.5418	<0.005*
		TT	40	41	38	52	0.22	0.0813–0.5915	9.8701	<0.005*
		CT+TT	92	94	47	64	0.12	0.0454–0.3063	23.9247	<0.005*
		C	64	33	61	42	Ref			
		T	132	67	85	58	0.68	0.4333–1.0534	3.0060	0.0830
TSLPR	rs36139698	total	55	100	84	100				
		CC	6	11	4	5	Ref			
		CT	11	20	46	55	6.27	1.5072–26.1067	7.4432	0.0064*
		TT	38	69	34	40	1.34	0.3489–5.1622	0.1842	0.6678
		CT+TT	49	89	80	95	2.45	0.6580–9.1144	1.8811	0.1702
		C	23	21	54	32	Ref			
		T	87	79	114	68	0.56	0.3181–0.9792	4.1890	0.0407*
	rs36177645	total	119	100	47	100				
		AA	13	11	3	6	Ref			
		AG	39	33	18	38	2.00	0.5062–7.9025	1.0034	0.3165
		GG	67	56	26	56	1.68	0.4427–6.3874	0.5926	0.4414
		AG+GG	106	89	44	94	1.80	0.4884–6.6245	0.7978	0.3717
		A	65	27	24	26	Ref			
		G	173	73	70	74	1.10	0.6359–1.8886	0.1087	0.7416
	rs36133495	total	119	100	90	100				
		CC	24	20	16	18	Ref			
		CT	61	51	43	48	1.06	0.5028–2.2235	0.0216	0.8830
		TT	34	29	31	34	1.37	0.6156–3.0382	0.5926	0.4414
		CT+TT	95	80	74	82	1.17	0.5792–2.3571	0.1892	0.6636
		C	109	46	75	42	Ref			
		T	129	54	105	58	1.18	0.8002–1.7488	0.7100	0.3995
IL‐7R	rs12516866	total	123	100	46	100				
		GG	49	40	22	48	Ref			
		GT	61	50	20	43	0.73	0.3580–1.4895	0.7497	0.3866
		TT	13	10	4	9	0.69	0.2006–2.3408	0.3663	0.5450
		GT+TT	74	60	24	52	0.72	0.3653–1.4286	0.8770	0.3490
		G	159	65	64	70	Ref			
		T	87	35	28	30	0.80	0.4776–1.3386	0.7253	0.3944
	rs1053496	total	89	100	28	100				
		CC	11	12	3	11	Ref			
		CT	19	22	8	28	1.54	0.3375–7.0630	0.3159	0.5741
		TT	59	66	17	61	1.06	0.2642–4.2245	0.0060	0.9380
		CT+TT	78	88	25	89	1.18	0.3035–4.5504	0.0547	0.8150
		C	41	23	14	25	Ref			
		T	137	77	42	75	0.90	0.4466–1.8049	0.0916	0.7622
Patients smoking for ≤5 years:										
TSLP	rs10043985	total	77	100	55	100				
		AA	65	84	45	82	Ref			
		AC	5	7	9	16	2.60	0.8172–8.2725	2.7607	0.0966
		CC	7	9	1	2	0.21	0.0245–1.7356	2.5304	0.1117
		AC+CC	12	16	10	18	1.20	0.4791–3.0243	0.1558	0.6930
		A	135	88	99	90	Ref			
		C	19	12	11	10	0.79	0.3595–1.7336	0.3481	0.5552
	rs2289276	total	113	100	58	100				
		CC	45	40	13	22	Ref			
		CT	56	49	32	56	1.98	0.9300–4.2071	3.1906	0.0741
		TT	12	11	13	22	3.75	1.3820–10.1758	7.1085	0.0077*
		CT+TT	68	60	45	78	2.29	1.1117–4.7203	5.1827	0.0228*
		C	146	65	58	50	Ref			
		T	80	35	58	50	1.83	1.1582–2.8758	6.7904	0.0092*
	rs3806933	total	98	100	42	100				
		CC	6	6	17	41	Ref			
		CT	52	53	6	14	0.04	0.0116–0.1432	32.7314	<0.005*
		TT	40	41	19	45	0.17	0.0570–0.4932	11.6898	<0.005*
		CT+TT	92	94	25	59	0.10	0.0342–0.2687	25.2719	<0.005*
		C	64	33	40	48	Ref			
		T	132	67	44	52	0.53	0.3164–0.8989	5.6410	0.0175*
TSLPR	rs36139698	total	55	100	39	100				
		CC	6	11	4	10	Ref			
		CT	11	20	15	39	2.05	0.4632–9.0330	0.9071	0.3409
		TT	38	69	20	51	0.79	0.1994–3.1261	0.1137	0.7360
		CT+TT	49	89	35	90	1.07	0.2813–4.0815	0.0102	0.9195
		C	23	21	23	29	Ref			
		T	87	79	55	71	0.63	0.3237–1.2347	1.8171	0.1777
	rs36177645	total	119	100	37	100				
		AA	13	11	6	16	Ref			
		AG	39	33	16	43	0.89	0.2875–2.7486	0.0418	0.8379
		GG	67	56	15	41	0.49	0.1586–1.4832	1.6534	0.1985
		AG+GG	106	89	31	84	0.63	0.2224–1.8051	0.7389	0.3900
		A	65	27	28	38	Ref			
		G	173	73	46	62	0.62	0.3563–1.0694	2.9898	0.0838
	rs36133495	total	119	100	57	100				
		CC	24	20	13	23	Ref			
		CT	61	51	31	54	0.94	0.4209–2.0913	0.0243	0.8761
		TT	34	29	13	23	0.71	0.2786–1.7883	0.5413	0.4619
		CT+TT	95	80	44	77	0.86	0.3984–1.8352	0.1617	0.6876
		C	109	46	57	50	Ref			
		T	129	54	57	50	0.84	0.5404–1.3212	0.5461	0.4599
IL‐7R	rs12516866	total	123	100	16	100				
		GG	49	40	7	44	Ref			
		GT	61	50	8	50	0.92	0.3112–2.7083	0.0240	0.8768
		TT	13	10	1	6	0.54	0.0607–4.7764	0.3175	0.5731
		GT+TT	74	60	9	56	0.85	0.2974–2.4369	0.0901	0.7641
		G	159	65	22	69	Ref			
		T	87	35	10	31	0.83	0.3763–1.8339	0.2112	0.6459
	rs1053496	total	89	100	23	100				
		CC	11	12	4	17	Ref			
		CT	19	22	7	31	1.01	0.2411–4.2570	0.0003	0.9858
		TT	59	66	12	52	0.56	0.1521–2.0562	0.7798	0.3772
		CT+TT	78	88	19	83	0.67	0.1920–2.3368	0.3989	0.5276
		C	41	23	15	33	Ref			
		T	137	77	31	67	0.62	0.3046–1.2559	1.7873	0.1813

* *p* < 0.05, Ref = Reference allele.

Out of three *TSLPR* SNPs studied, only rs36139698 showed a significant association with smoking. The frequencies of “CT” genotype and “T” alleles were found to be more than sixfold higher in long‐term smokers at both the phenotypic and genotypic levels when compared those of the “CC” genotype and “C” phenotype references (OR = 6.27, CI = 1.507–26.107, *p* = 0.0064 and OR = 0.56, CI = 0.318–0.979, *p* = 0.0407, respectively). However, rs36139698 showed no association with short‐term smokers and controls. The genotype distribution of rs36139698 was 11% CC, 20% CT, and 69% TT in nonsmokers and 5% CC, 55% CT, and 40% TT in long‐term smokers. The “T” allele frequency distribution was 79%, 68%, and 71% in nonsmokers, long‐term smokers, and short‐term smokers, respectively (Table 4). An association was observed between the *TSLPR* SNPs rs36177645 and rs36133495 with both short‐term and long‐term smokers (Table 4). *TSLPR* rs36177645 had the following genotype frequency distributions: 11% AA, 33% AG, and 56% GG in nonsmokers; 6% AA, 38% AG, and 56% GG in long‐term smokers; and 16% AA, 43% AG, and 41% GG in short‐term smokers. Subjects carrying the *TSLPR* rs36177645 variant showed more similar phenotypes within the smoker subgroups than those in nonsmoker controls (Table 4). In addition, *TSLPR* rs36133495 had the following genotype frequencies: 20% CC, 51% CT, and 29% TT in nonsmokers; 18% CC, 51% CT, and 29% TT in long‐term smokers; and 23% CC, 54% CT, and 23% TT in short‐term smokers. The rs36133495 phenotype distribution was more similar among the different smoker subgroups when compared to nonsmoker controls (Table 4).

Finally, we investigated the potential association between *IL7R* SNPs and cigarette smoking based on duration of smoking. We observed no significant correlations with smoking behavior for both rs12516866 and rs1053496. *IL7R* rs12516866 showed the following genotype distribution: 40% GG, 50% GT, and 10% TT in nonsmokers; 48% GG, 43% GT, and 9% TT in long‐term smokers; and 44%, 50%, and 6% in short‐term smoker. However, the phenotype distribution was 65% G and 35% T in long‐term smokers and 70% G and 30% T in nonsmokers. Phenotype B for this SNP showed a genotype distribution of 40% GG, 50% GT, and 10% TT in nonsmokers and 44% GG, 50% GT, and 6% TT in smokers. Phenotype G was observed in 65%, 70%, and 69% of nonsmokers, long‐term smokers, and short‐term smokers, respectively, while the mutant phenotype T was observed in 35%, 30%, and 31%, respectively (Table 4). By contrast, the respective genotype distributions of the *IL7R* rs1053496 SNP for nonsmokers, long‐term smokers, and short‐term smokers were 12%, 11%, and 17% for the “CC” genotype, 22%, 28%, and 31% for “CT,” and 66%, 61%, and 52% for “TT” (Table 4). In addition, the respective phenotype distributions for nonsmokers, long‐term smokers, and short‐term smokers were 23%, 25%, and 33% for the “C” reference allele and 77%, 75%, and 67% for the “T” mutant allele (Table 4).

### 3.4 Association between *TSLP*, *TSLPR*, and *IL7R* SNPs and daily cigarette consumption

To investigate the association between daily cigarette consumption and genetic variations in *TSLP* and its receptors, smokers were categorized into the following two subgroups according to smoking frequency: heavy smokers, who consumed ≥20 cigarettes per day (about one pack; termed group A) and moderate smokers, who smoked <20 cigarettes daily (termed group B). Table 5 displays the genotypic distributions of the selected SNPs in either group A or group B relative to the entire control group. Two of the three *TSLP* SNPs analyzed showed statistically significant associations with smoking in both smokers subgroup (categories A and B) relative to nonsmokers. The first *TSLP* SNP, *rs10043985*, had the following respective genotype distributions for nonsmokers and groups A and B: 84%, 82%, and 76% for the “AA” reference allele; 7%, 18%, and 22% for heterozygous “AC”; and 9%, 0%, and 2% for double mutant “CC.” Notably, the double mutant “CC” genotype showed a clear association with group A smokers (*p* = 0.0075), whereas the heterozygous “AC” genotype showed more than fourfold higher correlation with group B smokers when compared to the “CC” homozygous reference genotype (OR = 3.90; CI = 1.279–11.895; *p* = 0.0120). The second SNPs is rs3806933, which showed a strong association with smoking in group A and B smokers relative to nonsmoker subjects (*p* < 0.005). The ‘T” allele was highly associated with group A smokers relative to controls (*p* = 0.0054) but did not appear to be associated with group B smokers (*p* = 0.1368) (Table 5). However, there were no significant associations between *TSLP* rs2289276 and both smoking groups. rs2289276 showed the following genotype distributions: 40% CC, 49% CT, and 11% TT in nonsmokers; 39% CC, 48% CT, and 13% TT in group A smokers; and 32% CC, 52% CT, and 16% TT in group B smokers (Table 5).

**Table 5:** Genotypic distributions of SNPs in smokers compared to entire controls based on daily cigarette consumption

Gene	SNP	Allele	Controls		≥20 Cig.		OR	95% CI	χ^2^	*p* value
			*N*	Percent	*N*	Percent				
Patients smoking ≥20 cigarettes/day										
TSLP	rs10043985	total	77	100	85	100				
		AA	65	84	70	82	Ref			
		AC	5	7	15	18	2.79	0.9584–8.0968	3.7689	0.0522
		CC	7	9	0	0	—	—	7.1584	0.0075*
		AC+CC	12	16	15	18	1.16	0.5057–2.6640	0.1238	0.7250
		A	135	88	155	91	Ref			
		C	19	12	15	9	0.69	0.3363–1.4059	1.0624	0.3027
	rs2289276	total	113	100	92	100				
		CC	45	40	36	39	Ref			
		CT	56	49	44	48	0.98	0.5445–1.7716	0.0036	0.9523
		TT	12	11	12	13	1.25	0.5020–3.1126	0.2303	0.6313
		CT+TT	68	60	56	61	1.03	0.5861–1.8079	0.0102	0.9196
		C	146	65	116	63	Ref			
		T	80	35	68	37	1.07	0.7136–1.6038	0.1068	0.7439
	rs3806933	total	98	100	68	100				
		CC	6	6	27	40	Ref			
		CT	52	53	11	16	0.05	0.0157–0.1409	37.5074	<0.005*
		TT	40	41	30	44	0.17	0.0611–0.4546	13.7746	<0.005*
		CT+TT	92	94	41	60	0.10	0.0380–0.2582	28.4268	<0.005*
		C	64	33	65	48	Ref			
		T	132	67	71	52	0.53	0.3378–0.8304	7.7475	0.0054*
TSLPR	rs36139698	total	55	100	75	100				
		CC	6	11	5	6	Ref			
		CT	11	20	35	47	3.82	0.9735–14.9748	3.9800	0.0460*
		TT	38	69	35	47	1.11	0.3096–3.9458	0.0238	0.8775
		CT+TT	49	89	70	94	1.71	0.4952–5.9341	0.7373	0.3905
		C	23	21	45	30	Ref			
		T	87	79	105	70	0.62	0.3464–1.0986	2.7156	0.0994
	rs36177645	total	119	100	54	100				
		AA	13	11	4	7	Ref			
		AG	39	33	20	37	1.67	0.4806–5.7800	0.6567	0.4177
		GG	67	56	30	56	1.46	0.4381–4.8341	0.3783	0.5385
		AG+GG	106	89	50	93	1.53	0.4758–4.9395	0.5185	0.4715
		A	65	27	28	26	Ref			
		G	173	73	80	74	1.07	0.6406–1.7989	0.0725	0.7877
	rs36133495	total	119	100	86	100				
		CC	24	20	19	22	Ref			
		CT	61	51	43	50	0.89	0.4346–1.8244	0.1006	0.7511
		TT	34	29	24	28	0.89	0.4018–1.9786	0.0796	0.7779
		CT+TT	95	80	67	78	0.89	0.4521–1.7554	0.1116	0.7383
		C	109	46	81	47	Ref			
		T	129	54	91	53	0.95	0.6406–1.4067	0.0673	0.7953
IL‐7R	rs12516866	total	123	100	33	100				
		GG	49	40	16	49	Ref			
		GT	61	50	14	42	0.70	0.3127–1.5798	0.7319	0.3923
		TT	13	10	3	9	0.71	0.1784–2.7992	0.2460	0.6199
		GT+TT	74	60	17	51	0.70	0.3250–1.5229	0.8005	0.3709
		G	159	65	46	70	Ref			
		T	87	35	20	30	0.79	0.4420–1.4284	0.5919	0.4417
	rs1053496	total	89	100	37	100				
		CC	11	12	5	14	Ref			
		CT	19	22	9	24	1.04	0.2779–3.9072	0.0037	0.9512
		TT	59	66	23	62	0.86	0.2684–2.7406	0.0672	0.7954
		CT+TT	78	88	32	86	0.90	0.2903–2.8063	0.0314	0.8594
		C	41	23	19	26	Ref			
		T	137	77	55	74	0.87	0.4625–1.6226	0.2011	0.6538
Patients smoking < 20 cigarettes/day										
TSLP	rs10043985	total	77	100	53	100				
		AA	65	84	40	76	Ref			
		AC	5	7	12	22	3.90	1.2787–11.8953	6.3165	0.0120*
		CC	7	9	1	2	0.23	0.0275–1.9574	2.1065	0.1467
		AC+CC	12	16	13	24	1.76	0.7317–4.2355	1.6167	0.2036
		A	135	88	92	87	Ref			
		C	19	12	14	13	1.08	0.5162–2.2650	0.0429	0.8360
	rs2289276	total	113	100	57	100				
		CC	45	40	18	32	Ref			
		CT	56	49	30	52	1.34	0.6625–2.7075	0.6635	0.4153
		TT	12	11	9	16	1.88	0.6743–5.2134	1.4737	0.2248
		CT+TT	68	60	39	68	1.43	0.7310–2.8122	1.1040	0.2934
		C	146	65	66	58	Ref			
		T	80	35	48	42	1.33	0.8370–2.1047	1.4521	0.2282
	rs3806933	total	98	100	43	100				
		CC	6	6	16	37	Ref			
		CT	52	53	4	9	0.03	0.0072–0.1151	35.6327	<0.005*
		TT	40	41	23	54	0.22	0.0740–0.6282	8.6147	<0.005*
		CT+TT	92	94	27	63	0.11	0.0392–0.3088	21.9330	<0.005*
		C	64	33	36	42	Ref			
		T	132	67	50	58	0.67	0.3995–1.1351	2.2141	0.1368
TSLPR	rs36139698	total	55	100	42	100				
		CC	6	11	3	7	Ref			
		CT	11	20	24	57	4.36	0.9180–20.7425	3.7495	0.0528
		TT	38	69	15	36	0.79	0.1745–3.5713	0.0945	0.7585
		CT+TT	49	89	39	93	1.59	0.3740–6.7750	0.4013	0.5264
		C	23	21	30	36	Ref			
		T	87	79	54	64	0.48	0.2508–0.9030	5.2578	0.0218*
	rs36177645	total	119	100	28	100				
		AA	13	11	5	18	Ref			
		AG	39	33	13	46	0.87	0.2591–2.8988	0.0540	0.8162
		GG	67	56	10	36	0.39	0.1138–1.3236	2.4004	0.1213
		AG+GG	106	89	23	82	0.56	0.1830–1.7388	1.0139	0.3140
		A	65	27	23	41	Ref			
		G	173	73	33	59	0.54	0.2947–0.9861	4.0929	0.0431*
	rs36133495	total	119	100	55	100				
		CC	24	20	9	16	Ref			
		CT	61	51	29	53	1.27	0.5235–3.0703	0.2771	0.5986
		TT	34	29	17	31	1.33	0.5094–3.4900	0.3443	0.5573
		CT+TT	95	80	46	84	1.29	0.5557–3.0003	0.3542	0.5517
		C	109	46	47	43	Ref			
		T	129	54	63	57	1.13	0.7180–1.7866	0.2869	0.5922
IL‐7R	rs12516866	total	123	100	26	100				
		GG	49	40	12	46	Ref			
		GT	61	50	13	50	0.87	0.3646–2.0773	0.0981	0.7541
		TT	13	10	1	4	0.31	0.0373–2.6424	1.2475	0.2640
		GT+TT	74	60	14	54	0.77	0.3297–1.8099	0.3542	0.5518
		G	159	65	37	71	Ref			
		T	87	35	15	29	0.74	0.3851–1.4255	0.8105	0.3680
	rs1053496	total	89	100	13	100				
		CC	11	12	1	8	Ref			
		CT	19	22	5	38	2.89	0.2985–28.0715	0.9000	0.3428
		TT	59	66	7	54	1.31	0.1458–11.6842	0.0570	0.8113
		CT+TT	78	88	12	92	1.69	0.2000–14.3186	0.2380	0.6256
		C	41	23	7	27	Ref			
		T	137	77	19	73	0.81	0.3192–2.0675	0.1907	0.6623

* *p* < 0.05, Ref = Reference allele.

To evaluate the association between *TSLPR* SNPs and smoking based on daily cigarette consumption, we examined the genotype distributions and allele frequencies for the three *TSLPR* SNPs. Results of the analysis are summarized in Table 5. Only rs36139698 was found to be associated with group A smokers relative to control subjects. We observed that the “CT” genotype had a fourfold higher association with smoking (OR = 3.82; CI = 0.974–14.975; *p* = 0.0460) in group A smokers compared to controls. In addition, rs36139698 showed no association with smoking at the phenotypic level; however, there was a protective association between allele T and smoking in group B smokers (OR = 0.48; CI = 0.251–0.903; *p* = 0.0218). For *TSLPR* SNP rs36177645, our analysis showed no significant differences between nonsmokers and group A smokers at both the genotype and phenotype levels; however, the “G” allele was strongly associated with smoking in the second category compared to control subjects (*p* = 0.0431). Additionally, *TSLPR* rs36133495 did not show any correlation with smoking in either group A or group B smokers (Table 5).

Finally, the two *IL7R* SNPs, namely, rs12516866 and rs1053496, showed no significant correlations with either group A or group B smokers (Table 5).

### 3.5 Structural and functional analysis of the P195L mutation in rs36139698

We examined the effects of the polymorphisms on the structure and function of TSLP and TSLPR. The *TSLP* SNPs selected in the current study were located in the promoter and 5'‐UTR regions and can influence *TSLP* expression in smokers by increasing promoter activity and enhancing transcription. However, *TSLPR* SNPs were located in the exon region and thus potentially affected TSLPR function. Only rs36139698 appeared to be associated with smoking in the Saudi population. Structural analysis showed that rs36139698 results in a proline 195 to leucine mutation. This residue is located on the surface of the extracellular domain of TSLPR close to a WS motif located between residues 200 and 204.

**Figure 1:**
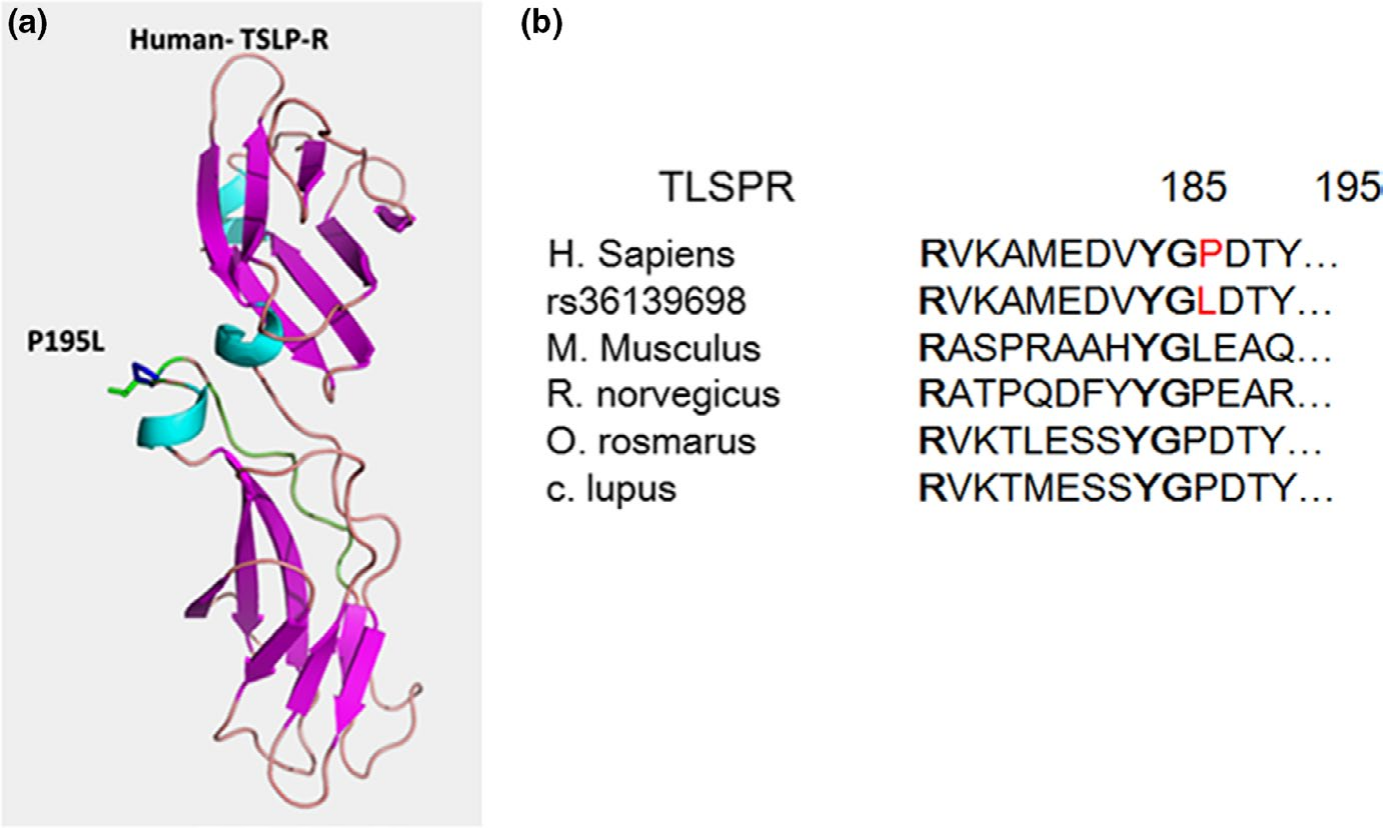
(a) Homology modeling of human TSLP receptor with P195L mutation. (b) Sequence alignment of TSLPR from different species near Proline 195. TSLPR rs 36139698 is located in the exon region and results in a proline 195 to leucine mutation

Sequence alignment of several TSLPRs (Figure 1) indicated that this proline residue is partially conserved and is replaced by a leucine in the mouse, similar to the rs36139698 variant. The P195L mutation is located on the surface and is accessible for hydrophobic interactions with TSLP, as observed in the mouse TSLPR structure.

From the X‐ray structure of the mouse TSLP‐TSLPR‐IL7α complex, this leucine is located in a loop at the interface and participates in hydrophobic interactions with TSLP. Substitution of proline by a leucine in the *TSLPR* human variant facilitates additional hydrophobic interactions that can further strengthen the binding with TSLP. No similar human protein structures are available. The stability of the P195L variant was assessed using CUPSAT stability prediction server. The variant has a predicted ΔΔG increase of 2.15 kcal/mol, thereby increasing the stability of the protein structure. This increased stability could increase the half‐life of the receptor and make it available for stronger interactions with TSLP, which in turn prolongs inflammation.

## 4 DISCUSSION

For a long period of time, scientific studies have not investigated the harmful effects of cigarette smoking on the oral cavity, lungs, and respiratory system. However, tobacco smoke has been later demonstrated to disrupt the lung and gingival epithelial barrier function (Semlali, Witoled, Alanazi, & Rouabhia, 2012), impair the innate immune system, and damage tissues by activating a variety of inflammatory immune cells. Semlali et al. provided substantial evidence that cigarette smoking (CS) promotes inflammation in the oral cavity and contributes to the development of gingival and periodontal disease by promoting the secretion of inflammatory cytokines (Rouabhia et al., 2017; Semlali, Chakir, Goulet, et al., 2011; Semlali, Chakir, & Rouabhia, 2011; Semlali et al., 2012). Genetic variants in the genes encoding these cytokines may contribute to susceptibility to smoking‐related diseases. Identifying the specific role of CS in acute inflammation is an important step towards elucidating the mechanisms underlying tobacco‐induced disease and can be used to develop novel therapeutic approaches for the management of diseases that afflict smokers. To our knowledge, the current study is the first to describe the association between variations in genes encoding *TSLP* and its receptors (TSLPR and IL7R) in smokers in Saudi Arabia, which has relatively high rates of smoking. The Saudi population has a considerably high incidence of respiratory diseases like asthma, COPD, periodontal diseases, oral cancers, and other tobacco‐related diseases. Thus, we analyzed and compared the frequencies of the *TSLP* and *TSLPR* polymorphisms from DNA isolated from smokers and healthy controls. Our findings highlight significant associations of *TSLP* and *TSLPR* SNPs, but not *IL7R* SNPs, with smoking behavior among Saudi smokers. Two *TSLP* SNPs, namely, rs10043985 and rs3806933, showed the strongest associations with smoking (*p* = 0.01 and *p* < 0.005, respectively). Furthermore, the SNPs rs3806933 and 10,043,985 were predicted to be implicated in proximal transcriptional regulation of *TSLP*. These polymorphisms are located in the promoter region of *TSLP* and could thus influence *TSLP* expression in smokers by increasing promoter activity and enhancing the binding of the transcription factor activating protein AP‐1 to the regulatory element of *TSLP* (Harada et al., 2009, 2011). This site is known to bind major transcription factors that regulate the expression of multiple inflammatory cytokines that play crucial roles in the pathogenesis of various airway diseases. Conversely, alterations in *TSLP* gene expression can directly affect the pathways involved in the development of inflammatory diseases.

Although the 5'‐UTR rs2289276 polymorphism was reported to be associated with higher risk of respiratory disease, such as asthma (Harada et al., 2011), it was not found to be associated with smoking in the population studied. Previous genome‐wide association studies have documented an association between the *TSLP* SNPs and risk for allergy diseases, such as asthma and airway hyperresponsiveness (Ferreira et al., 2014; Hirota et al., 2011; Torgerson et al., 2011). The principal role of the polymorphisms selected in the curent study in diseases related to smoking still unclear. Thus, the functional role of the TSLP polymorphism requires further investigation. Accumulating evidence has also supported the role of TLSP in promoting inflammation in the pathogenesis of infectious and autoimmune diseases, including oral cancer and asthma. We (Semlali, Jacques, Koussih, Gounni, & Chakir, 2010) and other authors (Hui et al., 2014; Lee et al., 2012) have previously demonstrated that TSLP expression is upregulated in asthma patients relative to healthy controls.

TSLPR and IL7R are the core subunits of the TSLP receptor and play crucial roles in TSLP signaling during inflammatory response. All three *TSLPR* SNPs studied herein are located in the exon region, and we hypothesized that the mutant TSLPR exhibits higher stability than the wild‐type TSLPR. In turn, this increased stability can prolong TSLP‐induced signal transduction and induce constitutive activation of the principal pathway of TSLP (Jak‐STAT pathway), causing inflammatory diseases as suggested recently by Mullighan et al (Ferreira et al., 2014). The results appear to support our hypothesis that the rs36139698 polymorphism, which corresponds to substitution of proline 195 into leucine and produces a TSLPR variant with a predicted ΔΔG increase of 2.15 kcal/mol, making the variant more stable than its wild‐type counterpart. This increased stability might increase the half‐life of the receptor making it available for interaction with TSLP maintaining the inflammation. P195L mutation located in the extracellular protein domain is able to bind to TSLP and it is close a WS motif, located between resisues 200 and 204 involved in receptor activation. Changes in the structural rigidity of this segment introduced by the P195L mutation may affect the function of the WS domain.

Consistent with previous studies, TSLPR gene polymorphisms were found to be correlated with increased susceptibility to atopic asthma in the Korean population( Yu et al., 2010) and with systematic lupus erythematous( Yu, Chun, Yun, Moon, & Chae, 2012). However, although several SNPs in *IL7R* have been associated with a wide range of diseases like liver disease in HIV/HCV infected patients (Guzmán‐Fulgencio et al., 2015) and sclerosis risk (Wu et al., 2016). Finally, our analysis demonstrated that smoking duration and consumption are correlated with the genotype frequencies of TSLP and TSLPR variants.

## 5 CONCLUSIONS

Although TSLP and TSLPR play crucial roles in inflammatory responses, the results of our study demonstrated a correlation between the TSLP and TSLPR variants and smoking behavior. Overall, our findings suggested that these genes can be utilized as diagnostic markers for all cigarette‐related diseases.

## CONFLICTS OF INTEREST

All authors declare no conflict of interest and all authors approved the manuscript.
